# The Association between Leukocyte and Its Subtypes and Benign Breast Disease: The TCLSIH Cohort Study

**DOI:** 10.1155/2020/3560593

**Published:** 2020-05-31

**Authors:** Yanqi Song, Xuena Wang, Liyan Huang, Yeqing Gu, Xingqi Cao, Jingzhu Fu, Hongmei Wu, Xiaojiang Li, Fanming Kong, Binxu Sun, Ruitian Sun, Qing Zhang, Li Liu, Ge Meng, Shunming Zhang, Yingjie Jia, Kaijun Niu

**Affiliations:** ^1^First Teaching Hospital of Tianjin University of Traditional Chinese Medicine, Tianjin, China; ^2^Nutritional Epidemiology Institute and School of Public Health, Tianjin Medical University, Tianjin, China; ^3^Health Management Centre, Tianjin Medical University General Hospital, Tianjin, China; ^4^Department of Toxicology and Sanitary Chemistry, School of Public Health, Tianjin Medical University, Tianjin, China; ^5^Tianjin Key Laboratory of Environment, Nutrition and Public Health, Tianjin, China; ^6^Center for International Collaborative Research on Environment, Nutrition and Public Health, Tianjin, China

## Abstract

Inflammation plays a crucial role in the formation of benign breast disease. Given the limited study to explore the association between leukocyte as an indicator of immune system and benign breast disease, we used data from a large cross-sectional study to investigate association between leukocyte and its subtypes and benign breast disease among women in the general population. The data were derived from baseline data of the Tianjin chronic low-grade systemic inflammation and health (TCLSIH) cohort study during 2014 and 2016. Breast thickness and nodules status were assessed by using ultrasonography. Leukocyte and its subtype counts were carried out using the automated hematology analyzer. Multiple logistic regression analysis was used to examine the association between leukocyte and its subtypes and prevalence of benign breast disease. In the present study, the prevalence of benign breast disease was 20.9%. After adjustments for potentially confounding factors, the odds ratios (95% confidence interval) for benign breast disease across lymphocyte quintiles were as follows: 1.00 (reference), 0.99 (0.82, 1.2), 0.85 (0.69, 1.04), 0.84 (0.68, 1.02), and 0.75 (0.61, 0.92) (*P* for trend = 0.002). An inverse association between lymphocyte counts and benign breast disease was found, but leukocyte and other subtypes have nothing to do with benign breast disease. Further prospective studies are needed to determine the findings.

## 1. Introduction

Breast cancer (BC) is a commonly diagnosed malignancy and the leading cause of cancer death in women worldwide [1]. Benign breast disease (BBD), the hallmark of which is epithelial proliferation, is a putative BC precursor [2]. BBD refers to a galaxy of pathophysiologic lesions resulting from progressive/regressive changes involving the component mammary structures, i.e., ducts, acini, stroma, and fat tissue [3]. The etiology of BBD is poorly characterized. Increasing evidence suggests that cancer-related inflammatory response plays a critical role in the development and progression of several malignancies. For instance, impairment of adaptive immune responses during chronic inflammation may favor tumor growth, angiogenesis, and cancer cell survival [4–8]. Previous studies have also indicated that inflammation pathways may be associated with BBD, from which early stage BC may develop [2, 9].

The primary response cells to inflammation are leukocytes. Leukocytes are the main building blocks of the immune system. The immune defense against tumors is largely a cell-mediated one and relies on the integrity of T cell subsets [9]. Meanwhile, B cells can also mount humoral immune response with antitumor effects [9]. Furthermore, different leukocyte counts reflect different levels of inflammation [10]. In past studies, researchers have found that different leukocyte counts are strongly associated with different types of cancer, such as endometrial cancer, lung cancer, endometrial cancer, and BC [11–13]. However, to the best of our knowledge, no previous studies have investigated the association between leukocyte and its subtypes and BBD in adult women.

Therefore, we designed a cross-sectional study to investigate how leukocyte and its subtypes are associated with the prevalence of BBD among women in the general population.

## 2. Materials and Methods

### 2.1. Study Population

The study sample was taken from participants in the Tianjin chronic low-grade systemic inflammation and health (TCLSIH) cohort, details of which have been published elsewhere [14]. This study conformed to the ethical guidelines of the 1975 Declaration of Helsinki. The protocols and procedures of the study were approved by the Institutional Review Board of Tianjin Medical University with the reference number of TMUhMEC 201430, and written informed consent was obtained from each participant.

**Figure 1:**
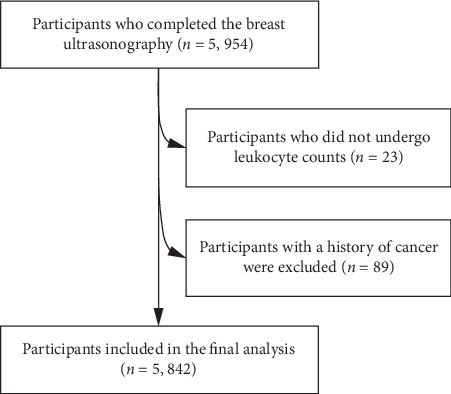
Flow diagram showing the selection of the study population.

During the research period, 5,954 participants, who received health examinations including body mass index (BMI), waist, a series of blood parameters, and breast ultrasonography and had completed questionnaires regarding their smoking and drinking habits and disease history over the course of January 2014 to December 2016, were recruited. We excluded participants who did not undergo leukocyte counts (*n* = 23). Participants with a history of cancer (*n* = 89) were also excluded. After these exclusions, the final cohort study population comprised 5, 842 participants (Figure 1).

### 2.2. Assessment of Leukocyte and Its Subtype Counts

Fasting blood samples were gained from all the participants by venipuncture of the cubital vein in the morning and immediately mixed with EDTA. Leukocyte and its subtype counts were carried out using the automated hematology analyzer XE-2100 (Sysmex, Kobe, Japan) and expressed as × 1, 000 cells/mm^3^. The test for blanks was ≤0.2 × 10^9^ cells/L; the intra- and interassay coefficients of variation (CV) were ≤2.0%; and the cross-contamination rate was ≤0.5%. In order to investigate how the leukocyte and its subtypes are associated with prevalence of BBD, we divided participants into quintiles based on leukocyte and its subtype counts.

### 2.3. Assessment of BBD

For the early screening and diagnosis of breast disease, both breasts were examined by breast ultrasound in the present study [15]. Breast ultrasonography was performed by trained sonographers using an iU22 ultrasonography system (Royal Philips) equipped with a 50 mm linear array transducer and a bandwidth of 7–12 MHz to measure breast gland thickness and status of breast nodule (position, size, and nature) [16]. All participants were asked to stay in the supine position and exposed breast. Arms are naturally lifted that bilateral breast and axillaries were fully exposed. Bilateral breasts were measured in turn. Measured indicators include breast thickness and breast nodule size. The lesions were classified as simple cyst, complicated cyst, fibrocystic changes, sonographically benign solid lesion (mass), and ductal ectasia. If an ultrasonographic image owned the features of more than one of any of the abovementioned lesions, then it was named as a mixed lesion [17]. Imaging results were also categorized into five levels using the Breast Imaging Reporting and Data System (BI-RADS) diagnostic category [18]. The intra- and inter-measure coefficients of variability (CV) were <2.9%.

### 2.4. General Examination

The anthropometric indicators (height, body weight, and waist circumference (WC)) were measured by well-trained investigators using a standard protocol. Waist circumference was measured at the umbilical level with subjects standing and breathing normally. The thresholds used to determine abdominal obesity were defined as WC ≥ 80 cm for Chinese women. BMI was calculated as weight/height^2^ (kg/m^2^) [19]. Obesity was defined as BMI ≥ 28 kg/m^2^ according to the criteria of the Working Group on Obesity in China (WGOC) [19].

The sociodemographic variables including age, disease history, family history as well as current medication, menstrual and reproductive history (age at menarche, parity, age at first full-term birth, and menopausal status), smoking (defined as ‘current smoker,' ‘ex-smoker,' and ‘nonsmoker'), and drinking status (defined as ‘everyday,' ‘sometimes,' ‘ex-drinker,' and ‘nondrinker') were noted by self-reported form on a questionnaire. If women were ex-smokers or ex-drinkers, they were asked age at which they had ceased smoking or drinking. Ex-smokers or ex-drinkers were defined as not smoking or drinking in a month prior to baseline, but used to be drinker and smoker. Occasional drinkers were defined as except for daily drinkers and drinking frequency more than one time in the past month prior to baseline. The history of surgery was assessed according to the responses to relevant questions and the personal health records.

All blood tests were performed after an overnight. As for lipids, total cholesterol (TC) and triglycerides (TG) were measured by enzymatic methods. Low-density lipoprotein (LDL) was measured by the polyvinyl sulfuric acid precipitation method and high-density lipoprotein (HDL) was measured by the chemical precipitation method. Levels of fasting blood glucose (FBG) were measured by glucose oxidase method. All these measurements were conducted by using appropriate kits on a Cobas 8000 automatic biochemistry analyzer (Roche, Mannheim, Germany). Blood pressure (BP) was measured twice in the right arm using an automatic device (TM-2655P, A&D Company, Ltd., Tokyo, Japan) after 5 minutes of rest in a seated position. The mean of two measurements was taken as the BP value.

### 2.5. Statistical Analysis

All statistical analyses in the present study were performed using the Statistical Analysis System 9.3 edition for Windows (SAS Institute). In this study, all continuous variables were not normally distributed by using the Kolmogorov-Smirnov test. The log transformation for those nonnormal distribution variables was applied before the statistical analysis. The continuous variables are presented as the geometric means and 95% confidence interval (CI) after logarithmic transforming, and categorical variables are shown as percentages. For analysis, the BBD statuses were used as dependent variables, and the quintiles of leukocyte and its subtype counts were used as independent variables. For characteristics analysis, differences among BBD statuses were examined using ANCOVA for continuous variables or using multiple logistic regression analysis for categorical variables after adjustment for age. Associations between different leukocyte and its subtypes counts and BBD were examined using logistic regression in three different models, and odds ratio (OR) and 95% CI were calculated. Analysis was performed without any adjustment in model 1; the analysis was adjusted for age and BMI in model 2; model 3 additionally adjusted for smoking status, drinking status, history of surgery, menopausal status, hypertension, hyperlipidaemia, diabetes, and the metabolic syndrome as well as for family history of CVD (cerebrovascular disease). The median value of each leukocyte subtype quintile was used to calculate the *P* values for linear trends. Interactions between different leukocyte subtypes counts, menopausal status, and confounders of BBD were tested by addition of cross-product terms to the regression model. The analysis applied a two-tailed significance test and considered *P* < 0.05 as an indication of statistical significance.

## 3. Results

Among 5,842 individuals, 1,221 participants (20.9%) met the criteria of BBD. The characteristics of the study participants according to no BBD and BBD status are shown in Table 1. Women with BBD were older than those without BBD. Subjects with BBD had lower BMI, WC, and SBP levels, were more likely to be everyday drinkers, and were less likely to be non-drinkers, and a higher proportion have had surgery (*P* < 0.05). No significant differences were observed between participants among TC, TG, LDL, HDL, DBP, FBG, smoking status, and history of CVD.

The crude and adjusted associations between leukocyte subtypes and BBD are presented in Table 2. After final multiple adjustment, the OR (95% CI) of BBD for leukocyte and its subtypes across the quintiles were as follows: leukocyte counts, 1.00, 1.01 (0.83, 1.22), 0.90 (0.73, 1.11), 0.94 (0.77, 1.16), and 0.92 (0.75, 1.14) (*P* for trend = 0.37); neutrophil counts, 1.00, 0.89 (0.73, 1.09), 0.88 (0.72, 1.08), 0.91 (0.74, 1.11), and 1.01 (0.82, 1.24) (*P* for trend = 0.71); monocyte counts, 1.00, 0.84 (0.70, 1.01), 0.84 (0.67, 1.04), 0.84 (0.69, 1.02), and 0.84 (0.69, 1.02) (*P* for trend = 0.05); lymphocyte counts, 1.00, 0.99 (0.82, 1.2), 0.85 (0.69, 1.04), 0.84 (0.68, 1.02), and 0.75 (0.61, 0.92) (*P* for trend = 0.002); neutrophil-to-lymphocyte ratio, 1.00, 0.93 (0.76, 1.14), 0.94 (0.77, 1.15), 1.11 (0.91, 1.36), and 1.08 (0.89, 1.32) (*P* for trend = 0.15), respectively. The test for interactions between menopausal status and BBD was not found to be significant after adjustment, and there were not significant interactions between the quintiles of leukocyte and its subtype counts and confounders of BBD in the final models (all *P* values for interaction >0.10).

## 4. Discussion

This study was designed to assess the association between leukocyte and its subtypes and BBD in a general women population. The results suggested that higher lymphocyte counts are independently associated with the lower prevalence of BBD. To our knowledge, this is the first large-scale study concerning the topic of lymphocyte counts and prevalence of BBD.

To date, only a nested case-control study has investigated the association between several inflammatory markers and BBD, and its results suggested that serum C-reactive protein (CRP) levels were independently associated with BBD, and adiponectin levels had an inverse association with BBD in the general women population [2]. Although leukocyte and its subtypes are simple and inexpensive markers of inflammation and associated with many diseases [20–22], no previous studies have assessed the association between leukocyte and its subtypes and BBD. The current study demonstrated that higher lymphocyte counts are independently associated with lower prevalence of BBD, whereas leukocyte and other subtypes did not demonstrate any association with BBD. More studies are needed to confirm whether these results can be observed in other populations.

Lymphocytes, especially tumor-infiltrating lymphocytes, play an important role in tumor-derived inflammatory responses [23–25]. Lymphocytes exert their antitumor activity by inducing cytotoxic cell death and inhibiting tumor proliferation [26]. Several studies have shown that increased infiltration of lymphocytes in tumor tissue predicted better survival outcomes in cancer patients [27, 28]. Therefore, low lymphocyte counts might result in an insufficient immunological reaction, which leads to inferior survival in multiple cancers [29, 30]. These mechanisms may partially explain our results. Further studies should be conducted to mechanistically describe the association between leukocyte and its subtypes and BBD.

The reliability and validity of these findings are strengthened by the population-based design, the large sample size, and the fact that we performed a detailed evaluation of an extensive set of potential confounders including sociodemographic, lifestyle, and clinically relevant variables. Previous studies have shown that surgery may affect the immunological and inflammatory response [31–33], resulting in a difference in leukocyte counts. Besides, the menopausal status can be an effect modifier or confounding factor for the association between breast tumor and WBC subtypes [34]. In order to avoid the influence of these variables, we adjusted them as confounding factors. Several limitations of the present study should be noted. First, this is a cross-sectional study, so we could not infer the causality between leukocyte subtypes and BBD. Therefore, further cohort studies and intervention trials should be conducted to confirm the association between leukocyte subtypes and BBD. Second, although numerous confounding factors were adjusted for during the analysis, the study cannot eliminate the potential effects of other unmeasured factors completely. Third, the use of oral contraceptives has been reported to influence the development of benign breast disease [35]. However, the present study did not collect the information on the use of oral contraceptives. Thus, the effect of oral contraceptives on the association between lymphocyte counts and benign breast disease cannot be ruled out. Finally, since we did not have specific pathological types of BBD, we are unable to assess the association between leukocyte subtypes and specific types of BBD. A more detailed study on different subtypes of BBD is needed to better understand the association observed between leukocyte subtypes and BBD.

In conclusion, the present study firstly showed that a higher lymphocyte count is associated with a lower prevalence of BBD. This finding suggests that lymphocyte counts might be a novel predictive factor or treatment target for developing BBD. Further prospective or clinical studies are necessary to confirm these preliminary findings.

## Figures and Tables

**Table 1:** Age-adjusted subjects characteristics by BBD status (*n* = 5, 842)^a^.

Characteristics	BBD		*P* value^b^
	No	Yes	
No. of subjects	4621	1221	—
Age (*y*)	47.9 (47.8, 48.0)^c^	48.5 (48.4, 48.6)	**<.0001**
BMI (kg/m2)	23.9 (23.8, 24.0)	23.6 (23.4, 23.8)	**<.001**
WC (cm)	80.3 (80.0, 80.5)	79.0 (78.6, 79.5)	**<.0001**
TC (mmol/L)	4.95 (4.92, 4.97)	4.95 (4.90, 5.00)	0.88
TG (mmol/L)	1.10 (1.08, 1.11)	1.09 (1.06, 1.11)	0.40
LDL-C (mmol/L)	2.83 (2.81, 2.86)	2.85 (2.80, 2.89)	0.62
HDL-C (mmol/L)	1.47 (1.46, 1.48)	1.48 (1.46, 1.50)	0.27
SBP (mmHg)	121.0 (120.6, 121.5)	119.4 (118.6, 120.3)	**0.001**
DBP (mmHg)	74.6 (74.3, 74.9)	74.2 (73.7, 74.8)	0.22
FBG (mmol/L)	5.07 (5.05, 5.10)	5.06 (5.02, 5.10)	0.61
Smoking status (%)	12.3 (11.4, 13.2)	11.3 (9.54, 13.3)	
Smoker	3.10	2.90	0.94
Ex-smoker	0.71	0.35	0.25
Non-smoker	96.2	96.7	0.68
Drinker (%)			
Everyday	0.58	1.32	**0.02**
Sometimes	34.5	37.2	0.23
Ex-drinker	5.67	6.94	0.21
Non-drinker	59.3	54.4	**0.03**
Menopausal status	53.1	43.7	0.11
History of surgery (%)	2.01	2.78	**0.03**
History of CVD (%)	6.15	4.10	0.75

BBD, benign breast disease; BMI, body mass index; WC, waist circumference; TC, total cholesterol; TG, triglycerides; LDL-C, low-density lipoprotein cholesterol; HDL-C, high-density lipoprotein cholesterol; SBP, systolic blood pressure; DBP, diastolic blood pressure; FBG, fasting blood glucose; CVD, cardiovascular disease.,^a^Analysis of covariance or multiple logistic regression analysis. ^b^Boldface indicates statistical significance (*P* < 0.05) (or appropriate value). ^c^Geometric mean (95% confidence interval) (all such values).

**Table 2:** Adjusted associations between categories of leukocyte and its subtypes and prevalence of benign breast disease^a^.

	Quintiles of leukocyte and its subtypes concentration (×10^9^/L)					*P* for trend^b, g^
	Level 1	Level 2	Level 3	Level 4	Level 5	
Serum leukocyte concentration (×10^9^/L, range)	3.92–4.71	4.71–5.40	5.40–6.10	6.10–7.40	≥7.40	
No. of subjects	1170	1241	1048	1180	1203	—
No. of BBD	258	272	207	242	242	—
Model 1^c^	1.00 (reference) d	0.99 (0.82, 1.20)	0.87 (0.71, 1.07)	0.91 (0.75, 1.11)	0.89 (0.73, 1.08)	0.18
Model 2^e^	1.00 (reference)	1.01 (0.83, 1.23)	0.90 (0.73, 1.10)	0.95 (0.78, 1.16)	0.93 (0.76, 1.14)	0.40
Model 3^f^	1.00 (reference)	1.01 (0.83, 1.22)	0.90 (0.73, 1.11)	0.94 (0.77, 1.16)	0.92 (0.75, 1.14)	0.37

Serum neutrophil concentration (×10^9^/L, range)	2.00–2.58	2.58–3.05	3.05–3.60	3.60–4.63	≥4.63	
No. of subjects	1193	1161	1101	1215	1172	—
No. of BBD	266	232	218	245	260	—
Model 1^c^	1.00 (reference)	0.87 (0.71, 1.06)	0.86 (0.70, 1.05)	0.88 (0.72, 1.07)	0.99 (0.81, 1.21)	0.82
Model 2^e^	1.00 (reference)	0.90 (0.74, 1.10)	0.89 (0.72, 1.08)	0.92 (0.75, 1.12)	1.04 (0.85, 1.26)	0.53
Model 3^f^	1.00 (reference)	0.89 (0.73, 1.09)	0.88 (0.72, 1.08)	0.91 (0.74, 1.11)	1.01 (0.82, 1.24)	0.71

Serum monocyte concentration (×10^9^/L, range)	0.20–0.27	0.27–0.32	0.32–0.38	0.38–0.48	≥0.48	
No. of subjects	2390	995	649	912	896	—
No. of BBD	532	203	129	181	176	—
Model 1^c^	1.00 (reference)	0.90 (0.75, 1.07)	0.87 (0.70, 1.07)	0.87 (0.71, 1.04)	0.85 (0.70, 1.03)	0.06
Model 2^d^	1.00 (reference)	0.87 (0.72, 1.04)	0.85 (0.68, 1.05)	0.85 (0.71, 1.03)	0.86 (0.71, 1.04)	0.06
Model 3^e^	1.00 (reference)	0.84 (0.70, 1.01)	0.84 (0.67, 1.04)	0.84 (0.69, 1.02)	0.84 (0.69, 1.02)	0.05

Serum lymphocyte concentration (×10^9^/L, range)	1.30–1.60	1.60–1.81	1.81–2.10	2.10–2.60	≥2.60	
No. of subjects	1185	1206	1114	1151	1186	—
No. of BBD	275	280	225	229	212	—
Model 1^c^	1.00 (reference)	1.00 (0.83, 1.21)	0.84 (0.69, 1.02)	0.82 (0.67, 1.00)	0.72 (0.59, 0.88)	**<0.001**
Model 2^e^	1.00 (reference)	1.01 (0.83, 1.22)	0.85 (0.70, 1.04)	0.83 (0.68, 1.01)	0.75 (0.61, 0.92)	**0.001**
Model 3^f^	1.00 (reference)	0.99 (0.82, 1.2)	0.85 (0.69, 1.04)	0.84 (0.68, 1.02)	0.75 (0.61, 0.92)	**0.002**

Serum neutrophil-to-lymphocyte ratio (range)	1.05–1.38	1.38–1.66	1.66–2.01	2.01–2.68	≥2.68	
No. of subjects	1173	1164	1168	1169	1169	—
No. of BBD	241	228	228	264	260	—
Model 1^c^	1.00 (reference)	0.94 (0.77, 1.15)	0.94 (0.77, 1.15)	1.13 (0.93, 1.38)	1.11 (0.91, 1.35)	0.09
Model 2^e^	1.00 (reference)	0.95 (0.77, 1.16)	0.96 (0.78, 1.17)	1.15 (0.94, 1.40)	1.12 (0.92, 1.36)	0.08
Model 3^f^	1.00 (reference)	0.93 (0.76, 1.14)	0.94 (0.77, 1.15)	1.11 (0.91, 1.36)	1.08 (0.89, 1.32)	0.15

^a^BBD, benign breast disease. ^b^Multiple logistic regression analysis. ^c^Model 1. Crude.^d^adjusted odds ratios (95% confidence interval) (all such values). ^e^Model 2, Age- and BMI-adjusted. ^f^Model 3, adjusted for age, body mass index, smoking status, drinking status, fasting blood glucose, systolic blood pressure, total cholesterol, triglycerides, high-density lipoprotein, low-density lipoprotein, surgery history, menopausal status, and family history of cardiovascular disease. ^g^Boldface indicates statistical significance (*P* < 0.05) (or appropriate value).

## Data Availability

The raw/processed data required to reproduce these findings cannot be shared at this time as the data also forms part of an ongoing study.

## Abbreviations

BC: Breast cancer
BBD: Benign breast disease
TCLSIH: Tianjin chronic low-grade systemic inflammation and health
BMI: Body mass index
CV: Coefficients of variation
WC: Waist circumference
WGOC: Working Group on Obesity in China
TC: Total cholesterol
TG: Triglycerides
LDL: Low-density lipoprotein
HDL: High-density lipoprotein
FBG: Fasting blood glucose
BP: Blood pressure
CI: Confidence interval
OR: Odds ratio
CVD: Cerebrovascular disease
CRP: C-reactive protein.
